# Determinants of health poverty vulnerability in rural areas of Western China in the post-poverty relief era: an analysis based on the Anderson behavioral model

**DOI:** 10.1186/s12889-024-18035-6

**Published:** 2024-02-14

**Authors:** Wenlong Wang, Kexin Chen, Wenwen Xiao, Jiancai Du, Hui Qiao

**Affiliations:** 1 School of Public Health, Ningxia Medical University, Yinchuan, China; 2 Key Laboratory of Environmental Factors and Chronic Disease Control, Yinchuan, China

**Keywords:** Vulnerability to health poverty, Andersen’s behavioral model, Multivariate logistic regression analysis, Shapley decomposition, China

## Abstract

**Background:**

Although China has eliminated absolute poverty, the effects of sickness still pose a threat to the prospect of returning to poverty in western rural areas. However, poverty governance extends beyond solving absolute poverty, and should enhance the family’s ability to resist risks, proactively identify the existence of risks, and facilitate preventive measures to reduce the probability of falling into poverty again. This study aimed to assess the health poverty vulnerability of rural households in western China and decompose its determinants.

**Methods:**

Based on survey data from 2022, the three-stage feasible generalized least squares method was used to calculate the health poverty vulnerability index. Then, Anderson’s health behavior theory model was extended to analyse various influencing factors using binary logistic regression, and the contribution of each influencing factor was decomposed using the Shapley index. Finally, Tobit regression and the censored least absolute deviations estimation (clad) method were used to test the model’s robustness.

**Results:**

A total of 5455 families in the rural Ningxia region of western China were included in the study. The health poverty vulnerability index of the sample population in 2022 was 0.3000 ± 0.2223, and families with vulnerability ≥0.5 accounted for 16.9% of the sample population. From the Anderson behavioral model, the three models including propensity, enabling, and demand factors had the best fit, and the AIC and BIC values were the smallest. The Shapley decomposition showed that the dimensions of the propensity factor, number of residents, age and educational level of the household head, and dependency ratio were the most important factors influencing vulnerability to health poverty. Tobit regression and the clad method proved the reliability of the constructed model through a robustness test.

**Conclusion:**

Rural areas still face the risk of becoming poor or falling into poverty owing to residents’ health problems. Health poverty alleviation should gradually change from a focus on treatment to prevention, and formulate a set of accurate and efficient intervention policies from a forward-looking perspective to consolidate the results of health poverty alleviation and prevent widescale poverty return.

## Introduction

Poverty, a global issue, has long hindered the development and progress of nations, with health-related poverty being particularly prominent. According to 2018 data from the World Bank, around 100 million people worldwide remain trapped in poverty due to illness among the 736 million individuals living on less than $1.90 per day [1, 2]. Poverty elimination remains one of the world’s most significant challenges. As a developing country that once had the largest rural poor population in the world, China attaches particular importance to poverty, shaping a unique poverty reduction strategy with Chinese characteristics and has made significant contributions to the global anti-poverty cause [3]. Specifically, after 8 years of sustained struggle, 832 counties nationwide with nearly 100 million poor people realized comprehensive poverty eradication, eliminating absolute poverty and regional poverty as a whole. By the end of 2020, China achieved the new century goal of completely eradicating extreme poverty. However, this does not mean that China’s poverty problem has been completely solved, nor does it mean that its anti-poverty and poverty-reduction efforts can be stopped after 2020. It is important to note that even if absolute poverty is eradicated, poverty is dynamic, and the risk of slipping back into poverty remains. Health problems and other unexpected shocks may cause nonpoor households or individuals to fall into poverty [4].

According to the Poverty Alleviation Office of the State Council of China, 42.3% of families registered as impoverished in China in 2017 either lived in poverty or returned to poverty because of illness [1]. According to the World Bank (2020), the COVID-19 pandemic will have a significant impact on poverty through multiple channels, including health and income setbacks. In particular, China’s entire population was lifted out of poverty by 2020 and was subsequently affected COVID-19. A recent study conducted in China showed that 23% of households that have overcome poverty since 2013 fear falling into poverty due to the impact of COVID-19. Additionally, 7.1% of households that had never been poor were expected to fall into poverty because of the pandemic [5], When a person’s health suddenly declines owing to illness or injury, this is referred to as a health shock. This can have a negative impact on both the individual and the well-being of their families, potentially leading to health poverty [6]. Individuals or families facing poverty due to health-related issues experience greater health risks and increased medical expenditures due to insufficient healthcare capacity. Additionally, their lower economic status limits their ability to invest in their health and face the financial challenges caused by illnesses, which can lead to a vicious cycle of poverty caused by illness and illness caused by poverty [7, 8]. Furthermore, research has shown that China’s health poverty is not only economic poverty but also physical and mental health, and has measured health poverty as a status of lacking physical health, mental health, and affordability of health care [9]. Although absolute poverty has been eliminated, relative poverty remains. Some people and families are still at risk of falling back into poverty, or poverty caused by illness. Therefore, it is particularly important to use the health poverty vulnerability index to prospectively identify potential victims of health poverty in rural areas, examine health poverty from multiple perspectives, and implement targeted interventions to reduce health poverty.

The World Bank first proposed poverty vulnerability, defined as the probability that a household will become poor in the future [10]. Vulnerability to health-related poverty refers to the probability that individuals or families will fall under a low welfare level after suffering from health-related risks, the risk of falling into poverty in the future, and the risk that the current state of poverty will continue [1]. Because vulnerability to poverty due to health problems is difficult to measure directly, it can be used as a risk factor or an early warning signal of a household’s likelihood of future poverty due to health-related problems [11].

The concept of vulnerability to health poverty is dynamic, as it connects the level of a household’s well-being to the potential health risks it may face in the future. A health poverty vulnerability perspective allows for the forward-looking and dynamic prediction of poverty associated with health problems and provides recommendations for targeted policy implementation. Some studies have shown that vulnerability to health poverty is more severe in less-developed regions, with a higher percentage of elderly households in rural areas of central and western China having a health poverty vulnerability of 0.5 or more than in economically developed eastern regions [12, 13]. According to previous studies, people living in rural areas of western China, especially those prone to chronic diseases, are at a high risk of health-related poverty. According to a survey conducted in Ningxia, a region in western China that has successfully overcome poverty, 41.5% of poverty cases are caused by diseases. The survey also revealed that disease was the main reason rural families had slipped back into poverty [11]. In addition, research shows that age, education level, health status, health insurance, total medical expenditure, two-week discomfort, and visit location significantly impact health poverty vulnerability [14]. Our research aims to examine various factors that contribute to the vulnerability to health poverty in rural areas of Ningxia, using Anderson’s health behavior model as a theoretical framework. We explored these factors from multiple perspectives to gain a comprehensive understanding of the issue.

Anderson’s health behavior model was developed in Western countries and has been widely tested. Anderson’s health behavior model, developed by Aday and Anderson, is widely used in Chinese healthcare as a reliable tool for studying health-service utilization. Recently, Anderson’s health behavior model has been used to explain medical decision-making behavior. Anderson’s health behavior model classifies the factors that may affect family health services into three dimensions. They are respectively predisposing factors (characteristics of people who have a tendency to use medical and health services before the onset of disease), enabling factors (personal resources and social resources), and need factors (preconditions and direct factors for using medical and health services) [15–17].

Predisposing factors inherent to family dynamics potentially affect vulnerable groups in health poverty, including limited health literacy, detrimental health beliefs, and sociocultural barriers. These factors hinder a comprehensive understanding of the severity of personal health issues, thereby affecting susceptibility to health poverty. Enabling factors, which are external determinants of the environment, encompass resources that facilitate or impede access to healthcare services. Vulnerable groups often encounter obstacles when attempting to utilize such services within these domains. Need factors primarily address the necessity and utilization of healthcare services, reflecting households’ proactive responses to health-risk shocks. While they often serve as direct predictors of healthcare usage, the fulfilment of these needs is frequently hindered by constraints imposed by predisposing and enabling factors, resulting in inadequate provision of medical requirements. The existing literature uses Anderson’s health behavior model to discuss health service utilization, but few studies have further explored the factors that affect health service utilization and the future health risk impact of households. However, studies have shown that predisposing, enabling, and need factors indirectly influence the utilization of medical and health services through economic poverty [18].

Most previous studies [19–21] have explored the impact of single factors, such as economy, education, and a poverty alleviation policy, on health poverty vulnerability. Few studies have used the Anderson behavioral model to comprehensively consider influencing factors from a multidimensional perspective, and research on multidimensional policy interventions is even more limited. Therefore, this study combines the three dimensions (predisposing, enabling, and need factors) of Anderson’s health behavior model to more carefully and accurately identify the risk of returning to poverty owing to diseases and health problems for the sample population who have been lifted out of poverty. This study comprehensively analyses the factors that affect vulnerability to health poverty and provides micro-level data support for adopting forward-looking measures to improve residents’ health, and for poverty reduction and sustainable development efforts to eliminate poverty. This study also enriches Anderson’s health behavior model.

In July 2017, Ningxia began to promote the alleviated health-related poverty. The continuous adjustment and implementation of health poverty alleviation policies help Ningxia realize the work of all poor areas out of poverty, but the completion of poverty does not represent the final victory of the work of poverty alleviation, while the latter part of the sustainable transition period is still spacing [22]. To consolidate the results of poverty eradication, it is essential to focus not only on the status quo but also on accurately identifying those who are potentially poor, and it is essential to use health poverty vulnerability as an indicator for identifying those who are potentially poor. In addition, appropriate support policies should be established for people with different characteristics in different regions to provide a basis for the establishment of accurate measures to prevent a return to poverty due to illness. Measuring health poverty vulnerability and exploring its determinants provides valuable micro-data to support poverty reduction, governance, and sustainable development efforts.

Accordingly, this study examines the current health poverty vulnerability of different types of rural families and the influencing factors by considering rural families in the Ningxia Hui Autonomous Region of China as the research object. Thus, this study addresses the following research questions: 1. What is the current vulnerability to poverty among rural households in western China? Households with high vulnerability to health poverty should be accurately identified to reduce the risk of falling into poverty and consolidate the gains from poverty eradication. 2. Reconstructing Anderson’s health behavior model to examine the factors influencing vulnerability to health poverty in rural western China and employing Shapley decomposition to identify the primary contributing factors. This study enriches the theory of poverty vulnerability from the perspective of health poverty and uses Anderson’s health behavior model as a theoretical framework to provide empirical research to curb the return of poverty due to illness after poverty alleviation in China, and to provide a theoretical basis for the work of health poverty alleviation in other regions of China and other developing countries.

## Methods

### Data sources

This study was funded by the National Natural Science Foundation of China, and the data were collected from the 2022 annual follow-up data of the Innovative Payment System to Improve Health Benefits project, a collaboration between the Ningxia Hui Autonomous Region Health Commission and Harvard University. To ensure accuracy, we conducted logical error correction on the database and removed any sample data with missing or unclear vital variables. From the remaining investigated households, 5455 families and 20,347 individuals were selected for this study. Our questionnaire had an effective rate of 97.25%, based on 5609 initial investigations. The investigation involved multistage stratified random sampling. Four counties were identified in the southern mountainous area of Ningxia were identified using random sampling. All administrative villages in each township of the four sample counties were divided into three levels according to the level of economic development, that is, good, medium, and poor. Using the random number table method, 40% of th villages were selected as sample villages, and 20–33 households were systematically selected as survey samples in each village. All permanent residents (residence time ≥ 6 months) were included in the survey. The survey was conducted face-to-face.

The sample size calculation formula for counting data in descriptive research is $n=\frac{u_{\alpha}^2\pi \left(1-\pi \right)}{\delta^2}$, The significance test level *α* = 0.05 is usually adopted, and the allowable error *δ* = 0.1 *π* is general. In 2019, the proportion of households in Ningxia that might fall into poverty in the future was 53.7%, means *π* = 53.7%. Thus, the required sample size is calculated to be about 332 households, The subjects included in this study meet the requirements of sample size.

### Model and variables

#### Health poverty vulnerability measurement

The theory of expected poverty vulnerability (VEP), which mainly uses Chaudhuri’s three-stage feasible generalized least squares (FGLS) method was adopted to measure rural families’ health vulnerability [23]. The specific steps are as follows:

First, to estimate the income equation, we assumed that the income of the rural population in period *t* + 1 is a function of individual characteristics in period *t*. We estimated a regression on the logarithm of future income and then perform ordinary least squares using the squares of the regressed residuals as income fluctuations

1 $$\ln {Y}_{it+1}=\beta {X}_{it}+{e}_{it}$$

where *Y*_*it* + 1_ represents the income level of the rural population in period *t* + 1, and *X*_*it*_ represents a set of observable variables of health poverty vulnerability that affect family income levels. This study combined health risk theory, capacity deprivation theory, and social capital theory to build an indicator system of health-poverty vulnerability from three dimensions: health risk, economic risk, and policy support risk. Owing to the heterogeneity of the sample population in different counties, townships, and villages, this study assumed that the income logarithm of the sample population in each village is generally distributed, but that the variance of income logarithm differens owing to differences in the individual characteristics of the sample population. Therefore, the residual square was regarded as an approximation of the income variance ${\hat{e}}_i^2$, and the residual square was used as the explained variable to construct the regression model of the residual square ${\hat{e}}_i^2$ on the sample population characteristic vector:

2 $${\hat{e}}_i^2=\theta X+{\eta}_i$$

The *Y*_*it* + 1_ and residual estimates can be obtained using the above two equations.

Second, using the heteroscedasticity structure as a weight, a weighted regression was run and the expectation and variance of the future income logarithm were estimated, as shown in Eqs. (3) and (4):

3 $$\hat{E}\left[\ln {Y}_i|{X}_i\right]={X}_i\hat{\beta}$$

4 $$\hat{V}\left[\ln {Y}_i|{X}_i\right]={\hat{\sigma}}_{ei}^2={X}_i\hat{\theta}$$

Finally, the poverty line was selected to estimate vulnerability to health poverty. At present, when defining the poverty line, Chinese scholars usually use an international poverty line of 2 or 3 US dollars, or China’s current poverty line of 2300 yuan (2010 constant prices). With China’s overall success in its fight against poverty, nearly 100 million people have been lifted out of poverty. At this stage, it is less meaningful using the absolute poverty line to measure poverty. Therefore, this study used the relative poverty line to measure relative health poverty. However, there was no unified definition standard for the definition of the relative poverty line, but research on the relative poverty line suitable for Ningxia was relatively limited. Some Chinese scholars believed that it can be set at 40 to 60% of per capita disposable income. An empirical study based on China’s Sichuan Province found that the relative poverty monitoring and early warning mechanism constructed using the per capita disposable income of rural residents as the relative poverty early warning judgment indicator has strong applicability [24–26]. This study drew on the standards of the European Union and OECD countries for delineating relative poverty lines, that is, most use 60% of per capita income as the cutoff for relative poverty lines. Therefore, this study selects 60% of the per capita income in Ningxia in 2022 as the standard for the relative poverty line [27–30]. In 2022, 60% of the per capita disposable income in the rural areas of Ningxia is 9202 yuan [31–33]. The data are from the Ningxia Statistical Yearbook 2022 [34] The value of health poverty vulnerability was distributed between 0 and 1. Zhang and Wan [4] found that prediction was more reliable when the vulnerability threshold was set at 0.5. Therefore, our study adopted a probability greater than 50% as the standard of vulnerability that is, household vulnerability is greater than 50%, which is considered vulnerable. A lognormal distribution is suitable for describing rural families. The method for calculating vulnerability to health poverty is shown in Eq. (5)

5 $${\hat{v}}_i=\hat{p}\left(\ln {Y}_i<\ln l|{X}_i\right)=\phi \left(\frac{\ln l-{X}_i\hat{\beta}}{\sqrt{X_i\hat{\theta}}}\right)$$

### Statistical method

Epidata 3.1 was used to input the survey data, and Stata 16.0 was used for data processing and statistical analysis. Two-sided *P* values less than 0.05 were defined as statistically significant. To summarize the characteristics of the sample, descriptive statistics were reported as mean, standard deviation (SD), and percentage. The Chi-square test was used to examine the association between vulnerability to health poverty and the independent variables. We defined households with a health poverty vulnerability index of more than 0.5 as vulnerable, and households with a vulnerability index of less than 0.5 as not vulnerable. Therefore, we used binary logistic regression to conduct multivariate regression analysis of the independent variables that have significant effects on vulnerability to health poverty. This study applies the concept of Shapley decomposition to poverty research to analyse the contribution of each factor to the vulnerability to healthy poverty. The analysis was conducted based on the three dimensions of Anderson’s health behavior model’s theoretical framework. This study included the Tobit model and censored least absolute deviations (clad) estimation for a comparative analysis to make the results more robust. According to the VEP theory, the health poverty vulnerability index has truncated discrete data at both ends, ranging from 0 to 1. This study employed the Tobit model and clad estimation for comparative analysis to avoid the bias induced by shortened data in the least squares method. Before data analysis, the assumption of multicollinearity was tested; we found no collinearity (VIF = 1.58).

Binary logistic regression was used to determine the probability (P) of a family falling into poverty and the occurrence ratio P(1-P). After logit transformation, $f(p)=\ln \left(\frac{p}{1-p}\right)$, the three constructed models primarily examine the impact of predisposing factors, enabling resources, and need factors on family health poverty vulnerability. Model 1 considers only predisposing factors, whereas Model 2 includes enabling factors in addition to predisposing factors. Model 3 incorporates all predisposing, enabling, and need factors, as follows:

Model 1: $$f(p)={\alpha}^1+{\beta}_1^1{x}_a+{\varepsilon}^1$$.

Model 2: $$f(p)={\alpha}^2+{\beta}_2^1{x}_a+{\beta}_2^2{x}_b+{\varepsilon}^2$$.

Model 3: $$f(p)={\alpha}^3+{\beta}_3^1{x}_a+{\beta}_3^2{x}_b+{\beta}_3^3{x}_c+{\varepsilon}^3$$.

where *f*(*p*) is the probability of household health poverty; *α* is the regression constant; *β* is the regression coefficient; *ε* is the random error; and *a*, *b*, and *c* represent the predisposing, enabling, and need factors, respectively.

Shapley decomposition based on regression model indicators, as proposed by Shorrocks (2013), has been extensively utilized in various economics fields to examine poverty and inequality. This decomposition allows for the examination of the determinants of the dependent variable and quantification of their contributions [35].

### Explanatory variable

As shown in Table 1, the dependent variable Y (1 = vulnerability, 0 = no vulnerability) is the household health poverty vulnerability, which is based on the VEP theory. In Tobit regression and the clad estimation, the dependent variable Y (health poverty vulnerability index) is used, and ranges from 0 to 1. Based on Anderson’s health behavior theoretical model, an independent variable (x) analysis framework composed of propensity, enabling and need factors was constructed, in which the household economic grouping of enabling factors was based on international standard economic quintiles.

**Table 1:** Classification and definition of independent variables

Variable		Definition	Reference
Predisposing factors	Gender	0 = Female	Female
		1 = Male	
	Age	Quantitative variable	
	Marital status	1 = Single	Single
		2 = Married	
		3 = Separated/Divorced	
		4 = Widowed	
	Ethnicity	1 = Han	Others
		0 = Others	
	Family size	1 = Single	Single
		2 = Small	
		3 = Big	
	Resident population	Quantitative variable	
	Dependency ratio		
	Household occupation	1 = Farmer	Rural nonfarm population
		0 = Ruralnonfarm population	
Enabling factors	Educational level	1 = No education	No education
		2 = Primary school	
		3 = Secondary school	
		4 = High school or more	
	Proportion of insured	Quantitative variable	
	Household incomes per capita		
	Household income grouping	1 = Low income level	Low-income level
		2 = Lower middle-income group	
		3 = Middle income group	
		4 = Upper middle-income group	
		5 = High income level	
	Whether it is a subsistence allowance	1 = Yes	No
		0 = No	
	Distance to nearest medical facility (km)	Quantitative variable	
	Percentage of major medical expenses		
Need factors	Families suffer from chronic disease types	Quantitative variable	
	Average household health score		
	Number of medical consultations per household		
	Number of hospitalized persons		
	Number of days of family hospitalization		
	Whether bills were incurred due to illness	1 = Yes	No
		0 = No	

### Sample characteristics

**Fig. 1:**
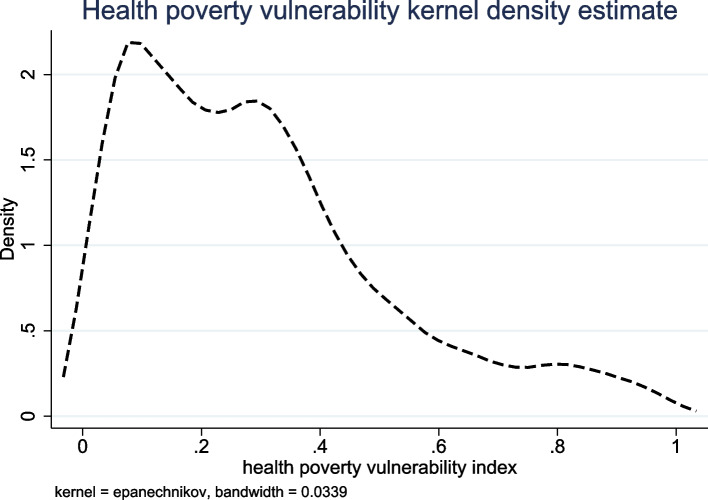
Kernel Density of Health Poverty Vulnerability

Results showed that the average health poverty vulnerability index of 5455 households was 0.3000 ± 0.2223. The kernel density of health poverty vulnerability is shown in Fig. 1. Among the participants, 4533 families had a health poverty vulnerability index of < 0.5, accounting for 83.1% of the entire sample. Moreover, 922 families had a health poverty vulnerability index of ≥0.5, accounting for 16.9% of the entire sample. This means that more than 16% of the families surveyed were at risk of falling into health poverty during the following year.

As shown in Table 2, a significant difference existed between vulnerable and non-vulnerable families for various variables. Specifically, health-poverty-vulnerable families are not of the Han ethnicity, have a non-subsistence allowance, are older, have a lower education level, have a large family size, have more resident population, have a higher dependency ratio, have members engaged in agricultural work, have lower household incomes per capita, have lower income levels, are closer to the nearest medical facility, have a higher percentage of serious disease insurance medical certificates, have more types of chronic diseases, have undesirable average household health scores, have more days of hospitalization, and have more bills incurred owing to illness compared to their non-vulnerable families.

**Table 2:** Description and comparison of the status quo of families with different health vulnerabilities [Mean ± *SD*, *N*(%)]

Variable	Total	Invulnerable families (*n* = 4533)	Vulnerable families(*n* = 922)	*P* value
Predisposing factors				
Age (in years)	55.17 ± 11.94	54.78 ± 11.61	57.05 ± 13.31	<0.001
Gender				
Male	3023(55.42)	2506(55.28)	517(56.07)	0.66
Female	2432(44.58)	2027(44.72)	405(43.93)	
Marital status				
Single	1710(31.35)	1413(31.17)	297(32.21)	0.48
Married	3451(63.26)	2884(63.62)	567(61.50)	
Separated/Divorced	47(0.86)	38(0.84)	9(0.98)	
Widowed	247(4.53)	198(4.37)	49(5.31)	
Ethnicity				
Han	2908(53.31)	2558(56.43)	350(37.96)	<0.001
Others	2547(46.69)	1975(43.57)	572(62.04)	
Family size				
Single	224(4.11)	222(4.90)	2(0.22)	<0.001
Small	3480(63.79)	3330(73.46)	150(16.27)	
Big	1751(32.10)	981(21.64)	770(83.51)	
Resident population	3.66 ± 1.79	3.17 ± 1.34	6.09 ± 1.74	<0.001
Dependency ratio	0.42 ± 0.37	0.40 ± 0.39	0.51 ± 0.24	<0.001
Household occupation				
Farmer	3784(69.37)	3201(70.62)	583(63.23)	<0.001
Rural nonfarm population	1671(30.63)	1332(29.38)	339(36.77)	
Enabling factors				
Educational level				
No education	1456(26.69)	1009(24.24)	357(38.72)	<0.001
Primary school	2148(39.38)	1772(39.09)	376(40.78)	
Secondary school	1436(26.32)	1278(28.19)	158(17.14)	
High school or more	415(7.61)	384(8.47)	31(3.36)	
Proportion of insured	0.99 ± 0.07	0.99 ± 0.07	0.90 ± 0.07	0.39
Household incomes per capita	16,421.60 ± 22,094.14	17,611.33 ± 23,484.40	10,572.31 ± 11,647.19	<0.001
Household income grouping				
Low-income level	1091(20.00)	855(18.86)	236(25.60)	<0.001
Lower middle-income group	1091(20.00)	841(18.55)	250(27.11)	
Middle-income group	1091(20.00)	914(20.16)	177(19.20)	
Upper middle-income group	1091(20.00)	932(20.56)	159(17.25)	
High-income level	1091(20.00)	991(21.86)	100(10.85)	
Whether it is a subsistence allowance				
Yes	2694(49.39)	2289(50.50)	405(43.93)	<0.001
No	2761(50.61)	2244(49.50)	517(56.07)	
Distance to nearest medical facility (km)	1.73 ± 1.86	1.77 ± 1.91	1.54 ± 1.59	<0.001
Percentage of serious disease insurance medical certificate	0.02 ± 0.10	0.02 ± 0.10	0.31 ± 0.09	0.02
Need factors				
Families suffer from chronic disease types	1.22 ± 1.36	1.18 ± 1.36	1.41 ± 1.36	<0.001
Average household health score	78.00 ± 10.46	78.16 ± 10.46	77.28 ± 10.44	0.02
Number of medical consultations per household	0.04 ± 0.15	0.04 ± 0.15	0.04 ± 0.12	0.76
Number of hospitalized persons	0.24 ± 0.92	0.23 ± 0.89	0.28 ± 1.04	0.12
Number of days of family hospitalization	6.94 ± 17.94	6.41 ± 18.17	9.57 ± 16.53	<0.001
Whether bills were incurred due to illness				
Yes	928(17.01)	735(16.21)	193(20.93)	0.001
No	4527(82.99)	3798(83.79)	729(79.07)	

## Results

### Binary logistic regression of health poverty vulnerability

From Table 3, a comprehensive observation of the three dimensions of Anderson’s health behavior model, the two information criteria (AIC and BIC), and pseudo-R-square (0.795) show that Model 3 has the best fitting degree, which is better than Models 1 and 2. The three binary logistic regression models constructed in this study passed the significance test and were statistically significant. Model 1, which included only the predisposing factors, showed significant results for age, resident population, dependency ratio, and household occupation. Model 2, which included both predisposing and enabling factors, showed that age, resident population, dependency ratio, and household occupation were significant as in Model 1. Ethnicity, educational attainment, and the proportion of critical illness outpatient medical certificates also contributed significantly. The results of Model 3 showed that predisposing factors, including age, ethnicity, resident population, dependency ratio, and household occupation significantly contributed to vulnerability to health poverty. Among these, the dependency ratio had the largest effect on the variation in vulnerability to household health poverty (*OR* = 1909.46), followed by the resident population (*OR* = 78.815). Increasing age and the occupation of the household head as a farmer also had positive effects on the change in vulnerability, with odds ratios of 1.295 and 6.366, respectively. Han ethnicity had a negative effect (*OR* = 0.566). Educational level and the percentage of serious disease insurance medical certificates showed significant values among the enabling factors. Improving the educational level had a negative effect on the change in health poverty vulnerability (*OR* < 1), whereas increasing the proportion of families with critical illness outpatient medical certificates had a positive effect on the change in health vulnerability to poverty (*OR* = 11.513).

**Table 3:** Results of binary logistic regression models for the health poverty vulnerability

Factor	Variable		Model 1			Model 2			Model 3		
			*OR* value	95%*CI*	*P* value	*OR* value	95%*CI*	*P* value	*OR* value	95%*CI*	*P* value
Predisposing factors	Age (in years)		1.253	1.225–1.281	<0.001	1.267	1.235–1.300	<0.001	1.295	1.259–1.332	<0.001
	Ethnicity	Han	1.060	0.794–1.416	0.69	0.621	0.444–0.869	0.005	0.566	0.397–0.806	0.002
		Others	REF	REF	REF	REF	REF	REF	REF	REF	REF
	Family size	Single	REF	REF	REF	REF	REF	REF	REF	REF	REF
		Small	3.511	0.422–29.215	0.42	2.602	0.330–20.546	0.36	2.600	0.318–21.261	0.37
		Big	2.345	0.264–20.830	0.44	1.585	0.179–14.057	0.68	1.589	0.174–14.488	0.68
	Resident population		27.850	21.032–36.878	<0.001	53.218	37.287–75.955	<0.001	78.815	52.856–117.525	<0.001
	Dependency ratio		339.565	161.085–715.795	<0.001	848.039	350.597–2051.275	<0.001	1909.446	720.719–5058.816	<0.001
	Household occupation	Farmer	3.574	2.638–4.843	<0.001	5.787	4.077–8.216	<0.001	6.366	4.402–9.205	<0.001
		Rural nonfarm population	REF	REF	REF	REF	REF	REF	REF	REF	REF
Enabling factors	Educational level	No education	REF	REF	REF	REF	REF	REF	REF	REF	REF
		Primary school				0.193	0.131–0.285	<0.001	0.182	0.121–0.273	<0.001
		Secondary school				0.050	0.029–0.086	<0.001	0.040	0.022–0.071	<0.001
		High school or more				0.009	0.003–0.022	<0.001	0.006	0.002–0.015	<0.001
	Household incomes per capita					1.000	1.000	0.98	1.000	1.000	0.76
	Household income grouping	Low-income level				REF	REF	REF	REF	REF	REF
		Lower middle-income group				1.095	0.700–1.714	0.69	1.119	0.700–1.789	0.64
		Middle-income group				0.816	0.506–1.316	0.41	0.764	0.465–1.258	0.29
		Upper middle-income group				0.874	0.535–1.427	0.59	0.861	0.519–1.429	0.56
		High-income level				0.762	0.405–1.434	0.40	0.716	0.376–1.364	0.31
	Whether it is a subsistence allowance	Yes				0.908	0.662–1.246	0.55	0.942	0.677–1.311	0.73
		No				REF	REF	REF	REF	REF	REF
	Distance to nearest medical facility (km)					0.939	0.856–1.030	0.18	0.905	0.818–1.001	0.05
	Percentage of serious disease insurance medical certificate					5.767	1.157–28.736	0.03	11.513	1.957–68.733	0.007
Need factors	Families suffer from chronic disease types								0.886	0.787–0.997	0.04
	Average household health score								0.986	0.971–1.002	0.09
	Number of days of family hospitalization								0.968	0.958–0.978	<0.001
	Whether bills were incurred due to illness	Yes							0.772	0.513–1.163	0.22
		No							REF	REF	REF
LR chi2			3629.50 ***			3857.48***			3939.970***		
Pseudo-R-square			0.7322			0.7782			0.795		
AIC			1343.21			1137.23			1062.742		
BIC			1396.05			1262.71			1214.641		

* *P* < 0.01 ** *P* < 0.05 ***P < 0.001, *REF* Reference

### Shapley decomposition of determinants of health poverty vulnerability

To quantify the contribution of these factors to health poverty vulnerability, we performed a Shapley decomposition of the indicators. The results of the Shapley decomposition in Tables 4 and 5 show the three dimensions of Anderson’s health behavior model and the roles of various influencing factors in health poverty vulnerability. As shown in Table 4, the predisposing, enabling and need factors contributed 0.744, 0.036, and 0.011, respectively, to health poverty vulnerability, accounting for 94.02, 4.59, and 1.39%, respectively. In addition, Table 5 presents the Shapley decomposition of the significant factors of health poverty vulnerability, showing that the contribution of the resident population is the highest, followed by age and dependency ratio, which contribute 74.44, 10.74, and 6.37%, respectively, to vulnerability to health poverty. In terms of educational level, household occupation, and ethnicity contributed 0.034, 0.011, and 0.011, respectively, to health poverty vulnerability, accounting for 4.35, 1.37, and 1.36% of health poverty vulnerability, respectively; however, the contributions of other variables are less than 1%.

**Table 4:** Decomposition of different dimensions of health poverty vulnerability

Dimension	Shapley value	Contribution (%)
Predisposing factors	0.744	94.02
Enabling factors	0.036	4.59
Need factors	0.011	1.39
Total	0.792	100.00

**Table 5:** Decomposition of the affect factors of health poverty vulnerability

Variable	Shapley value	Contribution (%)
Age (in years)	0.085	10.74
Ethnicity	0.011	1.36
Resident population	0.589	74.44
Dependency ratio	0.050	6.37
Household occupation	0.011	1.37
Educational level	0.034	4.35
Percentage of serious disease insurance medical certificate	0.001	0.15
Families suffer from chronic disease types	0.007	0.87
Number of days of family hospitalization	0.003	0.37
Total	0.792	100.00

### Robustness test

This study constructed a dummy variable to represent the degree of vulnerability to household health poverty. When the health poverty vulnerability of a households was higher than 50%, it was defined as 1; and when it was lower than 50%, it was defined as 0. To test the robustness of the factors that have a significant impact on vulnerability, Tobit regression and clad estimation were used for a comparative analysis of the health poverty vulnerability index. As shown in Table 6, among all the significant factors, except for the percentage of serious disease insurance medical certificates, the other influencing factors had a significant impact on the vulnerability index of health poverty. As there is little variation between the clad and Tobit estimates, it is reasonable to consider the clad estimated results as a set test for the Tobit model. It is clear that the results of the binary logistic model constructed based on Anderson’s health behavior model are robust.

**Table 6:** Tobit regression of health poverty vulnerability index compared with Clad estimates

Variable	Tobit	Clad
Age (in years)	0.00716*** (68.35)	0.00815*** (98.47)
Ethnicity	0.00747*** (3.65)	0.0105*** (6.57)
Resident population	0.117*** (195.03)	0.131*** (255.46)
Dependency ratio	0.196*** (64.83)	0.228*** (92.00)
Household occupation	−0.0447*** (− 21.32)	−0.0459*** (−28.19)
Educational level	−0.0460*** (−39.98)	− 0.0522*** (−56.96)
Percentage of serious disease insurance medical certificate	0.0111 (1.14)	0.0107 (1.35)
Families suffer from chronic disease types	−0.00620*** (−8.34)	− 0.00584*** (−10.29)
Number of days of family hospitalization	− 0.000755*** (−13.97)	−0.000850*** (− 17.07)
Constant	− 0.467*** (− 62.28)	−0.593*** (− 97.26)

* *p* < 0.05, ** *p* < 0.01, *** *p* < 0.001, Robust standard error in parentheses

## Discussion

In this study, the vulnerability of the rural areas of Ningxia based on the VEP theory, using 60% of Ningxia’s per capita disposable income as the poverty line, is higher than the 2015 China Financial Survey data and the health poverty vulnerability of empty-nester households in Shandong Province [36]. The reasons for the analysis are as follows, First, the subjects selected in this study are located in the former poverty zone in the rural areas of southern Ningxia, although all of them have been lifted out of poverty. However, compared to developed regions, basic health facilities are relatively backward, economic reserves are insufficient, and the ability to withstand health shocks and economic risks is relatively weak, resulting in higher vulnerability to health poverty. Secondly, the poverty criteria selected for this study were relatively high. Therefore, when comparing vulnerability to health poverty across regions, attention should be paid to poverty criteria, lines, and regional choices.

By comparing the basic situation of vulnerable and non-vulnerable households, the results show that the most vulnerable households are characterized by low income levels, lower education levels, and older heads of households. These households often engage in agricultural activities and have a lower overall economic status. Additionally, they reported poorer self-rated health status, longer hospital stays, a higher prevalence of chronic illnesses, and a higher percentage of critical illnesses among family members. These circumstances contribute to the inability of some families to pursue higher-paying and innovative jobs because of limited economic resources, lack of health knowledge, and poor health habits. Consequently, these households experience greater economic vulnerability and are more susceptible to health-related risks, which ultimately increases their likelihood of falling into poverty and the incidence of health poverty vulnerability. Contrary to our expectations, more vulnerable families were registered as subsistence households. However, this is consistent with the research of Sun and Duan [37], which shows that the subsistence allowance policy did not reduce rural households’ vulnerability to poverty but increased it. Most who received social assistance and participated in the subsistence allowance system were older, less educated, in poor health, and without steady work or income sources. Those meeting these statutory requirements can obtain considerable financial assistance from the current system. However, this has resulted in some users’ psychological dependence after getting help, as well as a lack of motivation to pursue a job and an optimistic outlook toward life, culminating in the ‘welfare dependency’ effect [38]. To some extent, this effect prevents rural households from participating in labour to improve their future health poverty status, and the subsistence security system only plays a limited role in the existing poverty of households, making it difficult to significantly reduce households’ vulnerability to health poverty. Our research also reminds developing countries and regions to some extent that some families with a high risk of health poverty should not only provide some ‘welfare’ to alleviate temporary health poverty, but also improve basic medical and health facilities, lead health education and publicity, actively promote employment, and improve people’s self-development ability and overall health status from various aspects. This reduces the risk of health poverty for families.

Based on Anderson’s health behavior model, our study concludes that vulnerability to health poverty should be comprehensively considered in multiple dimensions, such as propensity, enabling, and need factors. According to Klasen et al. [39], vulnerability to poverty is caused by the inability to manage risk shocks. This impact is caused not only by economic, health, education, and family issues but also by natural environmental variables, policy, and other equity considerations. Compared with the geographical detector model, Anderson’s health behavior model focuses more on exploring the impact of individual and family health behavior-level factors on vulnerability to health poverty. The geographical detector model is better for analysing the spatial relationship between environmental factors and health [40–42]. The unique advantage of Anderson’s health behavior model is that it has a complete indicator system and a theoretical analysis framework that can play a guiding role in the empirical analysis. Therefore, it has been widely used in the medical and health fields and has achieved good analytical results. It can incorporate multiple factors that may affect vulnerability to health poverty into a relatively mature and concise multidimensional analysis framework. Based on Anderson’s health behavior model, it is easy to accurately identify the risk factors that affect the health poverty vulnerability of families with different characteristics and provide practical and targeted suggestions from multiple perspectives. The results of the Shapley decomposition showed the moderating influence of the propensity factor on vulnerability to health poverty, followed by the enabling and finally, the need factor in Anderson’s theoretical model of health. In addition, the results of the Shapley decomposition of the influencing factors in each dimension showed that the Shapley value of each significant factor under Anderson’s health theory model was 0.792. Among them, the number of resident populations, age, and dependency ratio were the top three contributing factors, followed by education level, occupation of the head of household, and ethnicity; the remaining factors contributed relatively little.

Propensity factors are the demographic and social structural characteristics of families that influence their vulnerability to health poverty. This means that households with a higher vulnerability to health poverty are more likely to be negatively affected by such factors as higher average household age, limited access to healthcare, insufficient income generation capacity, or educational gaps. Households with a lower vulnerability to health poverty, on the other hand, are less likely to fall into health poverty in the future as a result of these variables. Ma, and other researchers arrived at similar conclusions. They pointed out that the demographic and social structure to cope with the impact of risk will greatly limit the possibility and feasibility of the family taking appropriate measures; therefore, the demographic and social structure is the main factor in avoiding current poverty and poverty vulnerability, and other external factors are only auxiliary [6]. Therefore, developing countries and regions should pay more attention to the timely understanding and assessment of the health status, specific circumstances, and needs of local people, especially families in rural areas, to ensure that they have access to quality basic health services. At the same time, such measures as the social security system and medical assistance program can be adapted to integrate the plan into policy formulation to ensure the effectiveness and sustainability of the policy, improve the ability of local families to cope with and manage the impact of health risks, and prevent families from experiencing serious and lasting health shocks. This reduces the likelihood of local households becoming poor because of health problems or becoming poor in the future because of health problems.

Among the factors that have a significant impact on health poverty vulnerability, the number of household residents is the most significant contributor, and the results show that vulnerability increases with the number of household members. Studies in developing countries, such as Ghana and Togo, have also concluded that the larger the family size, the higher the vulnerability to health poverty, and pointed out that the larger the family size will increase the degree of poverty and affect the vulnerability to poverty through health shocks and other channels [43, 44]. An increase in the resident population negatively affects per capita household consumption by changing the structure of household consumption. In the absence of universal welfare coverage, an increase in the number of permanent family residents also dilutes families per capita welfare level [45]. Large household sizes and large resident populations reduce the capacity of households’ capacity to cope with health-risk shocks and increase the likelihood of household poverty by affecting their per capita consumption levels and welfare.

In addition, our study found that the age of the household head and the dependency ratio of the family are important factors affecting the vulnerability of households to health poverty. As the age of the household head and family dependency ratio increase, the likelihood of a household falling into health poverty also increases. Jing [36] used the same methodology to obtain similar results and showed that the size of the labour force in a household and the age of the household head were important factors affecting vulnerability to poverty. As the age of the household head and the dependency ratio increase, the risk of health poverty also increases. In rural areas of western China, the head of the household, as the main source of income and the main labour force of a family, faces multiple risks, such as an increase in the risk of disease and a decrease in the source of economic income as the age of household head increases, which may ultimately increase the household probability of falling into health poverty in the future. Previous studies [46] have shown that adequate labour supply improves welfare and has a positive impact on poverty reduction. However, the increase in the dependency ratio in this study indicates that the household labour supply is insufficient, and the proportion of inactive people is higher. More households are at risk of insufficient economic income and lower per capita consumption, which reduces their ability to respond to health risk shocks and increases their vulnerability to health poverty.

Education provides the knowledge, skills, competencies, and values needed to pursue healthy, productive, and meaningful lives. The level of education also determines health status and number of healthy years of education. Lower levels of education indirectly lead to early departure from the workforce by impairing physical health, resulting in reduced lifetime earnings and economic savings [47]. According to our research, households whose heads have higher levels of education and non-farm employment are less likely to be poor because of health problems in the future. Education affects households’ vulnerability to health poverty by directly or indirectly influencing the length and type of work done by the educated, thereby influencing households’ income levels, consumption structures, and ability to cope with health risks and unexpected shocks, as Novignon and Ouadika, among others, have found in other developing countries [43, 48–50]. Therefore, it is recommended that policies to prevent poverty due to illness and the return to poverty include an education component, development of quality education, and promotion of access to lifelong education and training in rural areas through such measures as cost reduction and increased social benefits. Through the promotion of education to improve local people’s income-generating skills, rural inhabitants should be encouraged to engage in non-agricultural labour, and the number of healthy working years should be increased to improve their work income and economic reserves. With a focus on education and employment, we should fully recognize the role of high-quality education and non-agricultural work in poverty reduction, mobilize residents’ enthusiasm to invest in their own health, improve local residents’ ability to cope with health shocks, and reduce the likelihood of rural households falling into health poverty. This also serves as an empirical reference for many developing countries investigating ways to minimize health poverty [51, 52].

## Conclusions

Although absolute poverty has been completely eradicated in China, owing the unpredictable nature of the disease, families or individuals will fall into or return to poverty if the economic risk caused by the disease exceeds the ability of the family or an individual to bear it. Families in the rural areas of Ningxia, in which poverty was previously concentrated in western China, were selected as the research subjects. It also reflects the vulnerability to health poverty after total poverty alleviation and the health impact of COVID-19 and its influencing factors based on Anderson’s health behavior model analysis. From the perspective of health poverty alleviation, this study provides an empirical basis for improving the dynamic monitoring of poverty relapse prevention, further improving support mechanisms, and providing policy recommendations and a decision-making basis for continuously consolidating and expanding the achievements of poverty alleviation. At the same time, China has the best poverty alleviation policy among developing countries [2]. Therefore, this study can serve as a theoretical reference for developing countries in formulating poverty alleviation policies and measures for vulnerable groups and contribute to the implementation and execution of global poverty alleviation work.

The study found that some families in rural areas of western China are still at risk of falling into poverty because of health problems. Among the three dimensions of Anderson’s health behavioral theory model, propensity factors contribute the most to vulnerability to family health poverty, whereas demand factors contribute the least. Among the specific influencing factors, the number of permanent household residents, age of the head of household, family dependency ratio, and level of education of the household head were the main factors affecting vulnerability to health poverty. To consolidate the achievements of poverty alleviation in China, provide a reference for other developing countries on poverty reduction paths, prevent rural families from returning to poverty because of health problems, and provide policy recommendations on the driving factors of rural families’ vulnerability to health poverty.

Based on the population makeup, social structure, and health literacy of the local rural population, local government departments should identify critical groups for potential health poverty problems. They should formulate appropriate local policies to prevent and reduce disease poverty in the future and understand the focus of health policies. A focus on vulnerable families who are at high risk of developing health poverty due to the advanced age of the head of the household, the high number of permanent residents in the family, and the relatively high dependency on the family should be the priority of relevant departments in various regions. Therefore, building a comprehensive health promotion framework is crucial. This includes providing universal access to health knowledge for people of all ages, improving educational levels, and promoting non-agricultural employment. Access to education and training should also be provided in rural areas to enhance entrepreneurial and employability skills, and ensure sustainable economic security for the local population. Simultaneously, we will establish and enhance the social security system, which includes medical insurance, pension insurance, and unemployment insurance for rural residents; childcare services; and safety net systems for people with disabilities; improve community health care implementation; offer comprehensive health services; and boost residents’ motivation and enthusiasm to invest in health. Thus, the ability of local residents to cope with the impact of health risks improves, the likelihood of further economic poverty is avoided, and the possibility of falling back into health poverty is reduced.

Overall, China and other developing countries on the path to poverty alleviation should continue to maintain and improve the precision of policy implementation, adjust to local circumstances, practice sophistication, and establish a stratified and classified social assistance system. It is necessary to implement sustainable management policies with early detection, timely treatment, and healthcare, reduce the risk of health poverty, and consolidate and expand our achievements in poverty alleviation.

## Innovation of this study

This study examined the health poverty problem of families in rural areas of western China based on Anderson’s health behavior model, which provides a new research perspective to explore the factors that affect the vulnerability of families to health poverty. This study adds a health behavior model to conduct an overall assessment of family health poverty vulnerability, enriching the exploration of health poverty vulnerability from the perspective of a single economic structure. This study provides an analysis focused on family health behavior characteristics and recommendations for targeted interventions based on relevant family health behavior factors. Finally, this research is crucial for preventing the return to poverty in rural areas of western China and solving the vulnerability problem of health poverty.

## Data Availability

The data that support the findings of this study are available from Ningxia Medical University, but restrictions apply to the availability of these data, which were used under license for the current study and so are not publicly available. Data are, however, available from the submitting authors upon reasonable request and with permission of Ningxia Medical University.
